# Parent Perspectives of Co-Occupations in Neonatal Intensive Care: A Thematic Review of Barriers and Supports

**DOI:** 10.1177/15394492241271220

**Published:** 2024-08-19

**Authors:** Sydnee G. Stovall, Rylie G. George, Madelyn T. Lara, Kyra O. Gainous, Riqiea F. Kitchens, Claudia L. Hilton

**Affiliations:** 1 The University of Texas Medical Branch, Galveston, USA

**Keywords:** neonate, co-occupation, systematic review, caregivers, occupational engagement

## Abstract

**Background:**

Co-occupations within the neonatal intensive care unit (NICU), which include parenting activities, such as bathing, feeding, diapering, comfort care, and bonding for attachment, are consequential for optimal infant development.

**Objectives:**

This thematic systematic review examines supports and barriers for facilitating co-occupations between parents and infants in the neonatal setting.

**Methodology:**

A search of four databases (MEDLINE, CINAHL, PsycINFO, and PubMed) resulted in 20 studies that met inclusion criteria for data extraction.

**Results:**

Family-centered NICU design, good communication between parents and NICU staff, increased physical contact, parent involvement in caregiving, psychological wellness, parent education, peer support, and established parental roles are identified as supports to co-occupational engagement. Identified barriers include physical separation, loss of parental role, restrictions of the NICU environment, medical technology, role strain, psychological burden, lack of knowledge, and poor communication.

**Implications:**

Findings suggest that neonatal occupational therapy practitioners can facilitate parent-infant co-occupations by addressing barriers and augmenting existing supports.

## Plain Language Summary

Parent Perspectives of Co-Occupations in Neonatal Intensive Care: A Thematic Review of Barriers and Supports

This thematic review explores the current literature to provide information to health care practitioners on supporting caregivers of infants in the Neonatal Intensive Care Unit (NICU). In the NICU, infants are dependent on life-sustaining tasks, or occupations, that require the participation of both a caregiver and the infant, known as “co-occupations.” The intensity of the NICU environment is stressful to both the infant and caregiver, often limiting the engagement of co-occupations. These co-occupations, which include parenting activities, such as bathing, feeding, diapering, comfort care, and bonding for attachment, are consequential for optimal infant development. Therefore, occupational therapy must address the limitations of this engagement and best provide care that promotes reciprocal participation in these co-occupations. Findings indicate that barriers to parent-infant interactions include separation from the infant, disconnect from the parenting role, restrictions of the NICU, invasive medical technology, role conflicts, psychological impacts of the NICU, lack of medical knowledge and understanding, and poor communication with staff. Supportive factors include family-centered NICU designs, good communication with NICU staff, opportunities for physical contact, parental involvement, psychological support, parent education and support, and the establishment of parenting roles. The thematic analysis in this review informs the best approach for the facilitation of engagement between parents and their infants, by minimizing identifiable barriers and amplifying the presence of supportive factors.

## Literature Review

The neonatal intensive care unit (NICU) is a specialized unit that provides life-saving medical care for preterm, sick, or otherwise medically fragile infants (Harer & Moreno, 2019). The acute nature of this environment is critically detrimental to the harmony of a developing infant–parent dyad (Harer & Moreno, 2019). As neonatal occupational therapy strives to facilitate the infant-parent dyad within this complex environment, it is challenging to define the specific role of occupational therapy within the NICU interprofessional environment (Cardin, 2020; Craig et al., 2018).

The occupational therapy practitioner is an asset to the NICU interdisciplinary team which includes physicians, nurses, social workers, and others. As part of the multidisciplinary team, the occupational therapist provides expertise on neurodevelopmental positioning and caregiving while simultaneously facilitating interdisciplinary functions that support the acquisition of a parenting role (Cardin, 2020; Yu et al., 2020). In addition, occupational therapy seeks to maximize parent–infant bonding for optimal infant attachment and neurodevelopment by promoting positive caregiver and provider relationships (Craig et al., 2018). Occupational therapists also facilitate parenting by supporting caregiver involvement in simple infant care, promoting bonding opportunities, and providing meaningful activities for the dyad (Cardin, 2020; Price & Miner, 2009). However, most crucial, is the role of the occupational therapy team to analyze the barriers of parent-infant interaction and address such obstacles that impair co-occupational engagement (Craig et al., 2018; Price & Miner, 2009).

When discussing the occupational engagement of an infant, it is important to define co-occupational. Co-occupations exist between two parties who are dependent on each other to engage in a reciprocal task (Craig et al., 2018; Pierce, 2009). The occupations of both an infant and a caregiver are highly reliant on mutual participation; therefore, the term co-occupation is commonly used to describe parenting relationships (Craig et al., 2018; Pierce, 2009). Parent–infant co-occupations include feeding, bathing, diapering, soothing, comforting, bonding, and engaging with the infant. (Cardin, 2020). However, co-occupations only exist from the foundational occupation of parent role acquisition, an area critically disturbed by the NICU environment (Cardin, 2020).

It is important to clearly define the barriers and supports of the NICU to these co-occupations so that occupational therapy practitioners can best approach how to promote occupational engagement (Cardin, 2020). A competent occupational therapist needs to be aware of the barriers that decrease a parent’s perceived competence and well-being and capitalize on the supportive factors that foster the parent-infant relationship (Cardin, 2020). Occupational engagement not only provides parents with the opportunity to establish their new parental role but also supports the parent–infant bond necessary for healthy development (Günay & Coşkun Şimşek, 2021).

To date, a thematic analysis has not been published to clearly define factors that may influence the facilitation of parent and infant co-occupations in the NICU. Numerous qualitative studies have been published which have identified supportive and inhibiting factors of co-occupational engagement within independent NICUs (Bonner et al., 2017; Cardin, 2020; Fraga et al., 2019; Gibbs et al., 2016; Klawetter et al., 2019; Mäkelä et al., 2018; Nelson & Bedford, 2016; Ringham et al., 2022; Santos et al., 2019; Spence et al., 2023; Spinelli et al., 2016; Treherne et al., 2017; Yu et al., 2020). However, a systematic review of these findings has yet to be compiled and analyzed to form a generalized conclusion. This systematic review aims to identify the relevant supports and barriers that exist in creating successful parent-infant co-occupations within the NICU to further understand the most appropriate role for occupational therapy in facilitating these interactions.

## Method

### Study Design

This thematic systematic review interprets the perspective of NICU parents as qualitative themes of identifiable supports and barriers to parent-infant co-occupations in the NICU. The methods outlined to direct the data search for this review were conducted in line with the Preferred Reporting Items for Systematic Reviews and Meta-Analyses (PRISMA) statement for conducting and reporting systematic reviews (Page et al., 2021). The study was registered on Prospero on July 7, 2023, as CRN42023447763. An exhaustive search was conducted to obtain all relevant records available on the following online databases: MEDLINE, CINAHL, PsycINFO, and PubMed. An additional hand-search was conducted of online library resources accessed through Moody Medical Library at the University of Texas Medical Branch to prevent the omission of relevant records.

### Search Strategy

The search was conducted on July 13, 2023, and included all records published prior to the search date. A keyword search conducted on all databases included the following terms: ([“NICU” OR “Neonatal Intensive Care”] NOT (“trauma” OR “mental health” OR “depression” OR “quality of life” OR “breastfeeding” OR “home” OR “transition” OR “covid” OR “coronavirus” OR “psychosocial” OR “behavior” OR “development” OR “outcomes” OR “protocol” OR “illness” OR “testing” OR “surgery” OR “stress” OR “death” OR “coping” OR “treatments” OR “disease” OR “palliative” OR “decision making” OR “procedure” OR “tube” OR “neonatology” OR “incidence” OR “risk” OR “exposure” OR “feasibility” OR “effectiveness” OR “pain” OR “transmission” OR “prevalence” OR “life sustaining” OR “rounds” OR “nutrition” OR “fluid” OR “variant” OR “discharge”)) and (“Co-Occupations” OR “Cooccupations” OR “Occupations”) and (“Parent Role” OR “Parent” OR “Parenting” OR “Motherhood” OR “Fatherhood” OR “Parenthood”) and (“Barrier” OR “Support” OR “Supporting”). Limitations were applied for peer-reviewed records on PubMed.

### Inclusion Criteria

For recency purposes, only records published after January 1, 2016, were included. Studies considered for inclusion collected data using qualitative or narrative methods to preserve the unique, vulnerable, and invaluable perspective of NICU parents. Because NICU parents are one part of the interdependent infant-parent dyad, their perspective of support and barrier is invaluable to increasing their co-occupational engagement with their infant (Umberger et al., 2018). For inclusion consideration, all studies were required to identify themes that suggested barriers or supports of NICU co-occupations by providing qualitative parental perspectives of engagement.

### Exclusion Criteria

To address promote recency, records published before January 1, 2016, were excluded. All systematic reviews, meta-analyses, student theses and dissertations, non-peer-reviewed journals, and expert opinions were excluded. Records that were not available in English were also excluded. Any record with intervention groups for substantial consideration of post-traumatic stress disorder, postpartum depression, COVID-19, or other mental health/social circumstances were excluded. All records of data narratives about transitioning home were excluded.

### Data Collection

**Figure 1.**
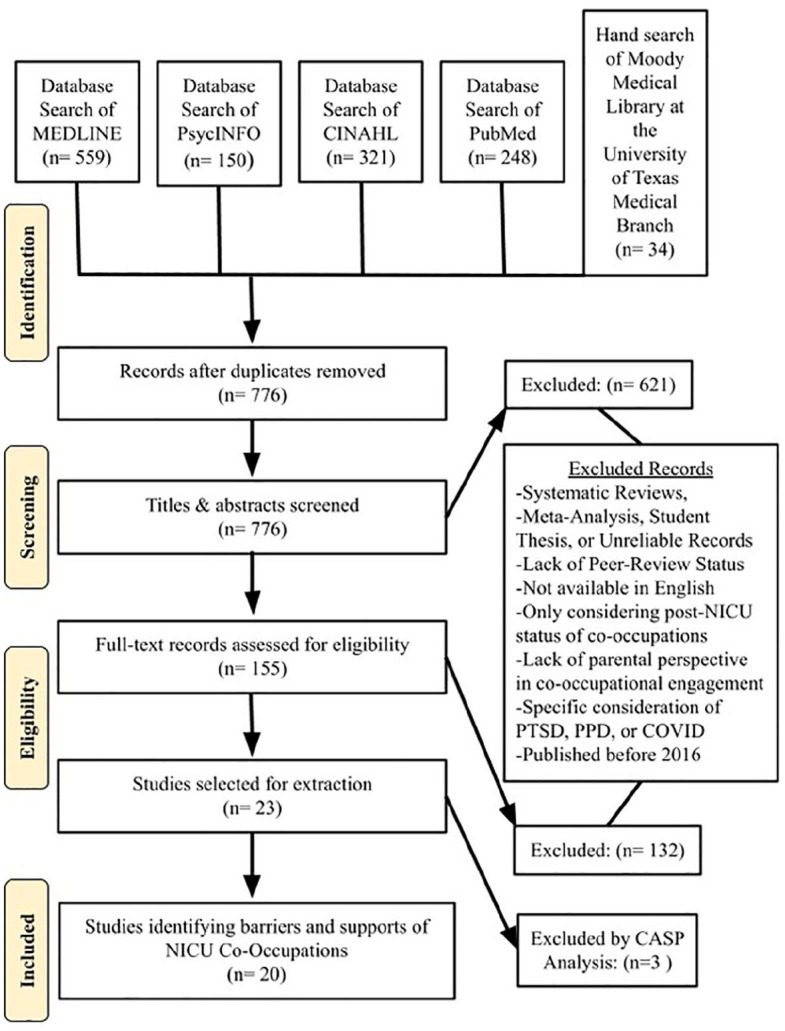
Flowchart of Search.

The flowchart in Figure 1 displays the phases of data collection. Data collection was completed in three phases using Covidence Systematic Review Management by three members of the review team. In Phase I, a total of 776 identified potential records were screened by title and abstract by a single reviewer, and duplicates were removed by Covidence. The review team used pre-determined exclusion criteria and majority rule to achieve interrater agreement during Phase I. In Phase II, a full-text review was completed of 165 records. Inclusion or exclusion of a record in the full-text phase required a vote by two unanimous members of the review team. In case of disagreement, the third reviewer assessed the record for selection. A total of 23 records were selected to be included for data extraction.

### Review Selection

Each record was screened for bias according to the Critical Appraisal Skills Program (CASP) Qualitative checklist as shown in *Appendix B* (Critical Appraisal Skills Programme, 2018). While 23 records were originally identified for inclusion, 3 additional records were discarded during the bias evaluation stage for inability to thoroughly meet inclusion criteria and associated ineligibility to apply the CASP Checklist. All three reviewers agreed that the ineligible records included studies or used methods that prevented thematic interpretation. After the review, 20 records met the inclusion criteria and were included for discussion.

### Thematic Analysis

The 20 records selected for analysis in this review present qualitative data to discuss the parental perspective of engagement in parent-infant occupations. Given the diversity in independent methodologies and the quantity of data, a narrative synthesis was composed to determine the identifiable themes of support and barriers. Data were extracted from each record to determine independent findings. Findings were then compared across studies using open coding to differentiate individual factors as either a support or a barrier. Within the support and barrier categories, themes were then defined to describe the specific factors governing overall engagement in co-occupations between NICU parents and their infants. The synthesis is shown in *Appendix A* for the narrative findings in each included independent study.

## Results

### Included Studies

Twenty studies were reviewed, all of which identified barriers and supports of infant and parent co-occupations throughout NICU admission. Of the twenty included studies, nineteen are characterized as qualitative, narrative, and phenomenological, studies utilizing semi-structured interviews, journal records, and observational methods of data collection for narrative synthesis. A singular study was classified as a qualitative ethnography (Ringham et al., 2022). The number of participants in each study varied between 6 to 614; however, all subjects were identified as a parent, either mother or father, of an infant admitted to the NICU. Six studies were conducted in NICUs across the United States. Other studies were conducted in hospital NICUs located in Canada (4), Brazil (2), Sweden (2), Australia, Turkey, Denmark, Finland, Italy, and China. All studies required the participant to be a parent of an infant admitted to the NICU; however, some studies utilized stricter criteria including length of NICU stay, age of parent, language, birthweight, gestational age, parenting experiences, and additional health issues, to control their study. Identified barriers and supports from each study were individually extracted to complete a thematic analysis. The characteristics and extracted data of the 20 included studies are shown in Supplemental Appendix A.

A thematic analysis was conducted to identify themes representing the seven supports and seven barriers of co-occupational engagement between NICU parents and infants. Individual barriers and supports were extracted from each study as seen in Supplemental Appendix A. All barriers were compiled to identify prevailing and encompassing themes as representative of occupational engagement. The methodology was repeated for all identified supporting factors. An exhaustive list of 14 themes was developed to represent the overarching supportive and inhibiting factors in NICU co-occupations. Barriers and supports of co-occupational engagement were identified, as shown in Tables 1 and 2, respectively.

**Table 1.** Thematic Analysis of Barriers: Barriers to Parent/Infant Co-Occupations in the NICU.

References	Physical separation from infant	Loss of parental role	Physical NICU environment and restrictions	Physical barrier of medical technology	Role strain	Psychological burden on parents	Lack of medical knowledge and poor communication
Antinora et al. (2023)	X		X			X	
Bonner et al. (2017)	X		X	X		X	
Campbell-Yeo et al. (2021)							
Cardin (2020)	X	X			X	X	
Dong et al. (2022)							
Fraga et al. (2019)	X	X	X	X	X		
Gibbs et al. (2016)		X	X	X	X	X	X
Günay & Coşkun Şimşek (2021)	X					X	
Klawetter et al. (2019)			X		X	X	X
Lilliesköld et al. (2022)	X	X				X	
Maastrup et al. (2018)				X		X	X
Mäkelä et al. (2018)	X						
Nelson & Bedford (2016)	X		X		X	X	X
Olsson et al. (2017)				X	X	X	
Ringham et al. (2022)		X	X		X	X	
Santos et al. (2019)	X	X		X		X	X
Spence et al. (2023)	X	X			X	X	X
Spinelli et al. (2016)	X	X	X	X			X
Treherne et al. (2017)	X	X	X	X		X	
Yu et al. (2020)	X		X			X	X

*Note.* Blank rows indicate references that mention supports but do not identify barriers of parent/infant co-occupations.

**Table 2.** Thematic Analysis of Supports: Supports of Parent/Infant Co-Occupations in the NICU.

References	Family-Centered NICU Design	Compassion and Communication of NICU Staff	Physical Contact with Infant	Parental Involvement in Infant Caregiving	Positive Psychological Wellness of Parents	Parenting Education and Peer Support	Establishing New Parental Roles
Antinora et al. (2023)	X						
Bonner et al. (2017)	X		X				
Campbell-Yeo et al. (2021)	X			X			
Cardin (2020)		X		X			
Dong et al. (2022)			X				X
Fraga et al. (2019)			X	X	X	X	X
Gibbs et al. (2016)		X	X	X		X	X
Günay & Coşkun Şimşek (2021)			X	X	X		
Klawetter et al. (2019)	X	X				X	
Lilliesköld et al. (2022)		X	X		X		X
Maastrup et al. (2018)			X				X
Mäkelä et al. (2018)	X	X	X	X			X
Nelson & Bedford (2016)	X	X		X		X	
Olsson et al. (2017)		X	X				
Ringham et al. (2022)							X
Santos et al. (2019)							
Spence et al. (2023)		X					
Spinelli et al. (2016)							
Treherne et al. (2017)	X	X	X	X			
Yu et al. (2020)	X					X	

*Note.* Blank rows indicate references which mention barriers, but do not identify supports of parent/infant co-occupations.

#### Barriers to Parent/Infant Co-Occupations

##### Physical Separation From Infant

Thirteen of the 20 studies discussed a barrier of physical separation between an infant in the NICU and their parent(s). Physical separation within the NICU environment can result in limited opportunities for interaction between parents and their infant (Cardin, 2020; Santos et al., 2019; Spence et al., 2023; Spinelli et al., 2016). The physical distance between the parent and the NICU can decrease opportunities for connection as well (Antinora et al., 2023; Fraga et al., 2019; Günay & Coşkun Şimşek, 2021; Mäkelä et al., 2018; Spence et al., 2023; Treherne et al., 2017; Yu et al., 2020). The wires and medical equipment attached to the infant also contribute to the barrier of physical separation (Bonner et al., 2017; Nelson & Bedford, 2016). In addition, the physical condition and medical status of the infant can create a further separation between that parent’s ability to interact and engage with their infant (Lilliesköld et al., 2022). These different components of physical separation create barriers to parent-infant bonding and impede the successful development of their emerging parenting role. This interferes with secure attachment, creating lifelong impairment in psychological well-being and emotional regulation.

##### Loss of Parental Role

Nine records indicated the loss of a parental role as a barrier to NICU co-occupations. This role disruption is described strongly as the inability of individuals to assume parenting responsibilities (Cardin, 2020) and the unmet expectations of parenting following birth (Gibbs et al., 2016). In addition, the caregiving role is shared with the NICU team (Fraga et al., 2019) with the primary caregiving responsibility held by the health care team (Santos et al., 2019; Spence et al., 2023). Two records report a loss of parenting role due to perceived judgment while being observed by the NICU staff (Lilliesköld et al., 2022; Spinelli et al., 2016). Other parental role loss was reported due to difficulties with breastfeeding (Ringham et al., 2022) and medical restrictions for parental participation (Treherne et al., 2017). Parents are less likely to engage with their medically fragile infant if they do not have feelings of responsibility in a caregiving role to the child, which often occurs within the NICU environment.

##### Physical NICU Environment and Restrictions

The physical NICU environment and associated policy restrictions can create a barrier to engagement in co-occupations, as identified by 10 records included in this review. Six studies identified policy and visitation restrictions as impairments to parental co-occupations (Antinora et al., 2023; Fraga et al., 2019; Gibbs et al., 2016; Ringham et al., 2022; Treherne et al., 2017; Yu et al., 2020) with one specifically referencing feeding restrictions as limiting co-occupational engagement and autonomy in parenting tasks associated with the feeding occupation. The particular design of the NICU with an associated lack of privacy for bonding and physical barriers to caretaking was identified by five studies (Bonner et al., 2017; Klawetter et al., 2019; Nelson & Bedford, 2016; Spinelli et al., 2016; Treherne et al., 2017). The lack of privacy and policies in a NICU environment can inflict doubt on parents hoping to have a larger role in NICU infant caretaking. This limits the volition of parents to assume parenting roles which is a necessary component of co-occupational engagement.

##### Physical Barriers of Medical Technology

Eight records pinpointed the physical barrier created by necessary medical equipment as a barrier to parenting co-occupations. Medical technology was described as a variety of wires, tubes, incubators, and other stabilizing medical equipment which limited physical interaction and caretaking through the duration of the NICU stay (Fraga et al., 2019; Gibbs et al., 2016; Maastrup et al., 2018; Olsson et al., 2017; Santos et al., 2019; Treherne et al., 2017). Wires and monitors reduced physical and emotional bonding as well as interfered with skin-to-skin contact time throughout the NICU stay (Bonner et al., 2017; Olsson et al., 2017; Spinelli et al., 2016). Being physically unable to touch their infants, parents felt disconnected from caregiving responsibilities and were less likely to engage in important co-occupations.

##### Role Strain

Role strain was established as a barrier to parent co-occupations in the NICU by eight records. All eight studies resolved that the conflict of sacrificing other responsibilities to spend time in the NICU interfered with the parent’s ability to participate fully in NICU parenting. Other obligations included time with other children, work, and other life activities outside the NICU environment (Cardin, 2020; Fraga et al., 2019; Gibbs et al., 2016; Klawetter et al., 2019; Nelson & Bedford, 2016; Olsson et al., 2017; Ringham et al., 2022; Spence et al., 2023). The associated distance from the hospital where the infant was admitted was included as an additional factor of role strain (Klawetter et al., 2019; Ringham et al., 2022; Spence et al., 2023). Mothers and fathers experiencing an increase in role strain, are less likely to pursue more occupational engagement opportunities with their infants, which is necessary to form critical parent-infant bonds.

##### Psychological Burden on Parents

The mental wellness of NICU parents was indicated as an occupational barrier by fifteen records selected for this review. Negative emotions such as shock, stress, anxiety, grief, frustration, and anger were specifically noted to be impairments of parental engagement (Antinora et al., 2023; Cardin, 2020; Gibbs et al., 2016; Klawetter et al., 2019; Ringham et al., 2022; Treherne et al., 2017). Negative emotions were further described as impairment due to feelings of insecurity in caring for a medically fragile infant and the associated fears of parenting a sick baby (Bonner et al., 2017; Günay & Coşkun Şimşek, 2021; Lilliesköld et al., 2022; Maastrup et al., 2018; Nelson & Bedford, 2016; Olsson et al., 2017; Santos et al., 2019; Spence et al., 2023; Yu et al., 2020). Parents who are suffering from more negative emotions and fear are less likely to find purpose in newly established NICU roles during their infant’s admission which decreases their overall engagement with the child.

##### Lack of Medical Knowledge and Poor Communication

Poor relationships and lack of adequate medical knowledge impaired engagement of co-occupations in the NICU by seven records. In one record, parents acknowledged feelings of exclusion by the medical team, impairing their responsibility as parents to care for their infants (Gibbs et al., 2016). In addition, parents identified that poor relationships and communication between NICU families and staff, as well as a perceived lack of parental permission, interfered with NICU co-occupations between parents and infants (Nelson & Bedford, 2016; Spinelli et al., 2016; Yu et al., 2020). An overarching attitude for this category was separation from parenting co-occupations due to a lack of medical knowledge or understanding of a medically fragile infant’s needs during NICU admission (Klawetter et al., 2019; Maastrup et al., 2018; Santos et al., 2019; Spence et al., 2023). Parents are less likely to engage with their infant in caregiving tasks when they do not feel they are properly equipped to handle the needs of their infant. Providing the parent with proper caregiver education can foster competence thus increasing the amount of parent-infant bonding that is essential for optimal physical, emotional, and social development after discharge from the NICU.

#### Supports to Parent/Infant Co-Occupations

##### Family-Centered NICU Design

Six studies positively identified the need for modernized NICU ergonomics to support and enhance the co-occupations between infants and their parents during admission. Technologies implemented to provide virtual connection after leaving the NICU, such as video recordings and video chats, helped to restore feelings of responsibility in infant caregiving, promoting overall co-occupational engagement even while physically separated from the infant (Antinora et al., 2023; Yu et al., 2020). Modernized use of wireless monitoring was identified as a means to increase physical contact with infants, restoring otherwise inhibited co-occupations between parent and infant (Bonner et al., 2017). Several records identified and emphasized a need for privacy and a modern approach to NICU design for private rooms to encourage parental responsibility and increase engagement in the caregiving role (Campbell-Yeo et al., 2021; Klawetter et al., 2019; Mäkelä et al., 2018; Nelson & Bedford, 2016; Treherne et al., 2017). In a NICU setting with a more ergonomic design, families are better equipped to establish roles that allow for engagement in parent-infant co-occupations. Occupational engagement is necessary to improve the physical and mental well-being of both the parent and the infant during and after the NICU.

##### Good Communication with NICU Staff

Positive relationships fostering compassion and good communication with NICU staff were noted by parents in nine of the included studies as a supportive factor in NICU co-occupations. When good communication and positive interactions were experienced by parents, there was a marked increase in overall involvement in decision-making, autonomy and parenting responsibility (Cardin, 2020; Gibbs et al., 2016; Klawetter et al., 2019; Nelson & Bedford, 2016). Compassion-centered communication and direct encouragement of parent involvement were also noted to increase co-occupational engagement and promote the participation of parents in infant caretaking throughout the infant’s admission (Lilliesköld et al., 2022; Mäkelä et al., 2018; Olsson et al., 2017; Spence et al., 2023; Treherne et al., 2017). With an increase of positive interactions within the NICU environment, parents feel more included in their role as the parents and are more likely to engage in co-occupations which can be preserved within the constraints of the NICU environment.

##### Physical Contact With Infant

Ten of the included studies identified the positive role of physical contact in promoting the overall engagement of parents with their NICU infants. Infants and parents experienced high levels of co-occupational engagement when provided with skin-to-skin or kangaroo care as an important role development in NICU caregiving in providing infants with bonding comfort which increased parental responsibility (Bonner et al., 2017; Dong et al., 2022; Fraga et al., 2019; Lilliesköld et al., 2022; Maastrup et al., 2018; Mäkelä et al., 2018; Olsson et al., 2017; Treherne et al., 2017). Even alternatives to physical contact with the infant, in terms of using warmth, scent, and voice recordings, parental engagement was found to be enhanced, despite limitations (Gibbs et al., 2016; Günay & Coşkun Şimşek, 2021). Physical contact with the infant is identified as an important element of providing co-occupational engagement between a NICU infant and their parents.

##### Parental Involvement in Infant Caregiving

One of the key components that many studies noted was the significance of parental involvement in infant caregiving in increasing co-occupations. Parents report feeling empowered and have increased confidence when they are involved in the small components of caregiving, such as changing diapers and taking the infant’s temperature (Cardin, 2020; Treherne et al., 2017). Furthermore, involvement in tube feeding or breastfeeding was identified as an important co-occupation to facilitate the development of parental role identity (Campbell-Yeo et al., 2021; Gibbs et al., 2016). Parents feel an increased sense of responsibility and self-confidence through infant caretaking (Günay & Coşkun Şimşek, 2021). Parenting roles can be supported by providing opportunities to share caregiving responsibilities between the parents and the NICU team when it is possible (Fraga et al., 2019). It is essential that parents feel they are provided with permission to be involved in caretaking tasks, and they are invited to be active participants by the NICU staff (Nelson & Bedford, 2016). Overall, parent–infant bonding can be increased when parents spend quality time with infants in the NICU and actively participate in infant care. (Mäkelä et al., 2018).

##### Positive Psychological Wellness of Parents

Several included studies stressed the importance of positive psychological wellness of NICU parents to facilitate co-occupation participation in the NICU. Parents with strong spirituality and internal motivation to participate are more likely to take an active parenting role, so health care providers need to provide safe spaces to encourage positive psychological well-being (Fraga et al., 2019). Furthermore, proper education and encouragement from NICU staff on co-occupations such as kangaroo care, can strengthen parent-infant bonding by increasing self-confidence, responsibility, and contentment in parenting roles (Günay & Coşkun Şimşek, 2021). NICU staff can enhance early skin-to-skin care by exhibiting positive emotions and giving affirmations, thus increasing parental reciprocal calmness and confidence (Lilliesköld et al., 2022).

##### Parenting Education and Peer Support

Five included studies identified the importance of parent education and the value of peer support as a positive contribution to co-occupations within the NICU. Several studies noted the importance of developing camaraderie with other parents in the NICU, as they understand each other’s shared experiences and emotions (Fraga et al., 2019; Gibbs et al., 2016). Klawetter et al., (2019) also explained the importance of peer support and the role hospitals should play in providing these connections. It is also important that the health care team provides adequate education on parental care, as prior parenting experience facilitates parental engagement in the NICU. One method to alleviate the lack of engagement due to poor education or perceived incompetence is to provide family education classes, which can also facilitate peer support (Yu et al., 2020). There are also programs implemented in some NICUs designed to empower and educate parents on caring for preterm infants, and overall allow them to be more active participants in their infant’s care based on the infant’s individualized goals (Nelson & Bedford, 2016).

##### Establishing New Parental Roles

Seven records identified the establishment of new parental roles as a support of overall infant-parent co-occupational engagement in the NICU. While many parents were stripped of traditional caregiving responsibilities while their infant was admitted, engagement could be preserved through a transition in their roles with adjustments in expectation and execution. Skin-to-skin, kangaroo care, and comfort care were identified as methods of supplemental parent and infant co-occupations which can be emphasized during a NICU admission to allow parents to feel involved in caretaking responsibilities (Dong et al., 2022; Fraga et al., 2019; Gibbs et al., 2016; Lilliesköld et al., 2022; Maastrup et al., 2018). Positive support for overall engagement was also identified in role development by emphasizing the importance of otherwise insignificant tasks, such as protecting the infant by self-awareness and adherence to hand hygiene and emphasizing participation in feedings by providing breast milk (Fraga et al., 2019; Mäkelä et al., 2018; Ringham et al., 2022). By stressing the importance of engagement in these secondary activities, parents were able to prioritize co-occupations by partaking in these roles to feel adequate in their parenting role.

## Discussion

To maximize family-centered care, the NICU multidisciplinary team must go beyond individual therapeutic interventions to best care for infants (Craig et al., 2018). Although the family-centered care approach is not performed exclusively by a singular discipline, occupational therapy interventions aimed at co-occupational engagement, directly promote and facilitate the development of the parent-infant bond (Craig et al., 2018). Neonatal therapy not only contributes to the comprehensive care of the infant for neurodevelopment but also functions to facilitate functional interactions of the parent-infant dyad. Although existing literature has identified the role of neonatal occupational therapy in promoting parent and infant co-occupations, the identifications made in this review inform the necessary therapeutic approaches that should be implemented (Cardin, 2020; Craig et al., 2018). This must begin by identifying disruptions to co-occupational engagement between the infant and the parent. The infant is dependent on their caregiver for nearly all occupational activities. However, the NICU is a non-traditional neonatal experience. Instead of parents providing this early caregiving, it is health care practitioners who are fulfilling the co-occupational role. This leaves many NICU parents with an inability to engage in the role of parenting, and infants unable to bond with their caregiver. It is through understanding how and where these barriers exist that occupational therapy must intervene. Occupational therapy has a unique health care role to advocate and promote wellness, concepts that are overtly apparent in supporting the infant-parent dyad. By providing education and implementing principles to minimize environmental barriers, the OT has a unique role in advocating for the parent-infant dyad within the NICU. Neonatal occupational therapy also works by fostering positive communication and psychological wellness in parents. It is the responsibility of occupational therapy to create space within the necessary elements of the NICU environment. While the medical needs of an infant will always take priority within the NICU environment, occupational therapy provides focus to ensure that parents fulfill their role as parents. From alternative comfort care methods, to including parents in feeding and positioning interventions, the occupational therapy team must be keenly aware to involve the parents in every occupation targeted by the occupational therapy team while the infant is cared for in the NICU.

While many roles exist for occupational therapy to limit barriers, other principle responsibilities fall to occupational therapy to facilitate supporting elements. This includes minimizing the stress of the neonatal environment by promoting ergonomic and family-centered care approaches, as well as implementing alternative methods of care which allow for increased interaction between the parent and infant for bonding. The occupational therapy team should facilitate as much physical contact as possible between the parent and infant during therapeutic sessions and invite parents to be active participants in caregiving duties. In addition, they should encourage the interdisciplinary team to do the same to nurture the parent-infant bond and increase the parent’s competence in the caregiving role. It is important for the occupational therapy team to utilize the limited quality time the parents have in the NICU with their infant to foster strong connections. The occupational therapy team should apply their training in mental health and wellness to deliver holistic, client-centered care to the parent and infant. Peer support groups and additional parent education are supplemental methods occupational therapy can use to increase self-efficacy and build camaraderie among NICU parents. The process of establishing new parental roles can be enhanced by involving the parent in co-occupations such as skin-to-skin contact and other seemingly insignificant tasks. The NICU team can greatly benefit from including occupational therapy, as the knowledge and holistic lens of occupational therapy can contribute to more comprehensive treatment for the parent and infant.

### Limitations

Several limitations in our systematic review exist, which pose a risk to the overall quality of our review. Our research looked specifically at qualitative studies as a means of understanding the specific barriers and supports identified from the perspective of parents. In doing so, we potentially limited our data pool, as we did not consider any quantitative studies or those collected from the perspective of the health care team. Given the nature of self-report data, we acknowledge the potential for subjectivity within the data. However, it was our intent to learn from the perspective of a parent and caregiver, as a participant in the co-occupational dyad, therefore subjectivity was necessary.

While all 20 studies were screened for bias, minute differences between individual NICUs and study interview formats could contribute as confounding variables in the available data sets. Participant bias and the confounding variables propose potential limitations in this thematic systematic review due to the sensitive nature of the subject discussed and the varying practices that may be implemented in different NICUs. We acknowledge that willing participants in individual studies are likely parents of medically stable infants, which limits the inclusion of parents with medically unstable or terminal infants. Each record is subject to bias from the assumed lack of inclusion of this perspective. In addition, several records located were unable to be translated into English. While our review includes a significant amount of diversity, other perspectives had to be excluded due to the inability to access English translations. It is also important to note that our research team decided to exclude all dissertations despite a plethora being available. We acknowledge that in doing so, we potentially eliminated data that could have contributed to our review.

Each individual record contributes limits to our review, by the risk associated with independent sampling bias. This bias is also present due to the qualitative nature of data sets. Many records included small numbers of participants, which may contribute to increased bias within individual studies.

Additional studies were excluded if COVID-19 or the mental health of the parents were a considerable component of the research. Although this review considers the mental well-being of parents as a factor that can be addressed by the occupational therapy practitioner, we excluded studies that solely measured the mental health of parents to consolidate our research for co-occupational engagement of the parent and infant. In doing so, this study may not fully disclose the relationship between the parent’s mental health and its effect on bonding and occupational engagement. While this criterion promoted the homogeneity of our review, our research neglects to reflect specific changes in supports and barriers as a result of post-pandemic society or mental wellness as identified in parents.

### Future Research

Future qualitative research should address the effectiveness of specific supports the magnitude of the hindrance created by specific barriers to parent-infant co-occupations directly, and the role of occupational therapy in addressing these. While this study evaluated the existing literature on the supports and barriers to parent-infant bonding and co-occupations, limited research exists on the benefits of the emerging role of neonatal occupational therapy, including how occupational therapy interventions contribute to developmental outcomes and impact the bond of the parent and infant. Future studies with a narrower focus on the value of having occupational therapy in the NICU setting will improve understanding of the scope and strengthen advocacy of the profession. Moreover, future studies directly addressing how co-occupations that occur in the NICU influence infant development will give practitioners further insight into the profound impacts occupational therapy has on patient well-being.

## Conclusion

Given the findings of this review, this study concludes that it is imperative for the occupational therapy team to support optimal infant development through co-occupational engagement by:

- utilizing therapeutic knowledge to increase parenting roles within occupational therapy intervention;
- implementing the use of supporting factors of engagement to better promote the infant-parent dyad; and
- limiting the presence of identified barriers to prevent additional disruption to parent–infant engagement.

## Supplemental Material

sj-docx-1-otj-10.1177_15394492241271220 – Supplemental material for Parent Perspectives of Co-Occupations in Neonatal Intensive Care: A Thematic Review of Barriers and Supports Supplemental material, sj-docx-1-otj-10.1177_15394492241271220 for Parent Perspectives of Co-Occupations in Neonatal Intensive Care: A Thematic Review of Barriers and Supports by Sydnee G. Stovall, Rylie G. George, Madelyn T. Lara, Kyra O. Gainous, Riqiea F. Kitchens and Claudia L. Hilton in OTJR: Occupational Therapy Journal of Research

sj-docx-2-otj-10.1177_15394492241271220 – Supplemental material for Parent Perspectives of Co-Occupations in Neonatal Intensive Care: A Thematic Review of Barriers and Supports Supplemental material, sj-docx-2-otj-10.1177_15394492241271220 for Parent Perspectives of Co-Occupations in Neonatal Intensive Care: A Thematic Review of Barriers and Supports by Sydnee G. Stovall, Rylie G. George, Madelyn T. Lara, Kyra O. Gainous, Riqiea F. Kitchens and Claudia L. Hilton in OTJR: Occupational Therapy Journal of Research
