# Supplementary material for: Parent Perspectives of Co-Occupations in Neonatal Intensive Care: A Thematic Review of Barriers and Supports
Source: OTJR (Thorofare N J). 2024 Aug 19;45(3):378–87. doi: 10.1177/15394492241271220 (PMC12130602; doi:10.1177/15394492241271220)
Supplement: sj-docx-1-otj-10.1177_15394492241271220 – Supplemental material for Parent Perspectives of Co-Occupations in Neonatal Intensive Care: A Thematic Review of Barriers and Supports [file sj-docx-1-otj-10.1177_15394492241271220.docx]

***Appendix A***

Data Extraction Evidence Tables

| **Author/Year** | **Study Design, and**  **Risk of Bias** | **Participants, Inclusion Criteria, & Study Setting** | **Intervention and Control Groups** | **Outcome Measures** | **Results (Including significance of findings)** |
| --- | --- | --- | --- | --- | --- |
| 1.  **Antinora et al., 2023**  DOI: 10.1097/JPN.0000000000000698 | **Study Design:**  Qualitative, Descriptive study; paper-based surveys before and after implementation of NeoConnect  **Risk of Bias:**  Moderate | **Participants:**  n= 24 parents of NICU infants  n= 68 NICU staff members  **Inclusion Criteria:**  Parent of baby admitted to NICU from less than a week to greater than a month  **Study Setting:**  Single-center Level 4 NICU | **NeoConnect program implemented in NICU:**  interdisciplinary team approach that included (1) playing recording of parents’ voice in incubator, during post nursing cares, or blood draws and (2) video chat program to participate in medical rounds, receive nursing updates, and meet with physicians  Narrative; Survey | ***Emerging Themes:***  **Pre-Implementation:**  1) Stress from being separated from baby  2) Desire to be more involved in infant’s care  **Post-Implementation**  1) Stress level  2) Involvement in Infant’s care  3) Strength of bond  4) Frequency of NICU visits  5) Difficulty with technology | **Results:**  *Barriers:*  1) Stress  2) Distance  3) Visitation restrictions  *Supports:*  1) Voice recordings made parents feel more involved, less stressed, and strengthened bond  2) Video chats made parents feel more involved, strengthened bond, and helped understand care plan  **Significance of Findings:**  Technology based programs may strengthen family-centered care in NICU |
| **Author/Year** | **Study Design, and**  **Risk of Bias** | **Participants, Inclusion Criteria, & Study Setting** | **Intervention and Control Groups** | **Outcome Measures** | **Results (Including significance of findings)** |
| 2.  **Bonner et al., 2017**  DOI: 10.1136/bmjinnov-2016-000145 | **Study Design:**  Qualitative, Descriptive study; Semi-Structured Interviews  **Risk of Bias:**  Low | **Participants:**  n= 6 parents with babies in NICU  n=7 nurses that cared for babies  **Inclusion Criteria:**   - Suitable parents determined by nurses in NICU based on emotional state and imposition of nurses’ time - Babies born at LBW or premature   **Study Setting:**  Single NICU | Narrative; Semi-Structured Interviews | ***Areas of Analysis:***  1) Current perceptions of the existing patient monitoring systems and their peripheral attachments  2) Future perceptions of a system which removes the wires between the sensor probe and the patient monitoring device | **Results:**  *Barriers:*  1) Seeing baby attached to wires elicited sadness, shock, and fear  2) Wires and monitoring systems impacted physical interaction and skin to skin contact due to fear  3) Wires reduced kangaroo care time and hindered positioning  *Supports:*  1) Wireless monitoring would improve kangaroo care and parent-infant bonding  **Significance of Findings:**  The appearance of infants in the NICU being connected to many wires and monitors may be stress-inducing for parents and reduce opportunities for physical bonding. |

| **Author/Year** | **Study Design, and**  **Risk of Bias** | **Participants, Inclusion Criteria, & Study Setting** | **Intervention and Control Groups** | **Outcome Measures** | **Results (Including significance of findings)** |
| --- | --- | --- | --- | --- | --- |
| 3.  **Campbell-Yeo et al., 2021**  DOI: 10.1097/JPN.0000000000000600 | **Study Design:**  Observational Cohort; Questionnaires and parent diaries  **Risk of Bias:**  Low | **Participants:**  n= 21 mothers  **Inclusion Criteria:**   - English speaking Mothers with infants with good prognosis - Longer than 5 day stay - Less than 10 days postnatal at the start of study.   **Study Setting:** Canadian NICU undergoing renovation from open-bay to single-family room style | **Open-Bay NICU**  (N=35 )  **Single Family Room NICU**  (N=36)  Narrative; Journaling and Questionnaires | All participants kept a diary to track time with infants and time engaged in infant caregiving.  Standard Questionnaires included Postpartum Depression Screening, Parental Stressor Scale, EQ-5D-5L, PTSD Checklist, Perceived Maternal Parenting Self-Efficacy, and Intolerance of Uncertainty Scale Questionnaire | **Results:**  *Supports:*  1) Single Family Room (SFR) NICU set-up increases overall participation in caretaking and comforting by the Father  2) SFR also showed increased participation of Mothers in pumping breast milk for the child  **Significance of Findings:**  Single-Family Room NICUs increase co-occupations for infant caregiving by both parents. |

| **Author/Year** | **Study Design, and**  **Risk of Bias** | **Participants, Inclusion Criteria, & Study Setting** | **Intervention and Control Groups** | **Outcome Measures** | **Results (Including significance of findings)** |
| --- | --- | --- | --- | --- | --- |
| 4.  **Cardin, 2020**  DOI:  10.5014/ajot.2020.034827 | **Study Design:**  Qualitative, Phenomenological study; Semi-Structured interviews  **Risk of Bias:**  Low | **Participants:**  n= 14 parents  **Inclusion Criteria:**   - Parents age 18-40 years of hospitalized infants at the time of the study   **Study Setting:**  Level 3 NICU with single family rooms in midwestern US | Narrative; Semi-Structured Interviews | ***Emerging Themes:***  1) Perceiving “they” vs. “I”  2) Maintaining proximity  3) Expressing emotions, values, and beliefs  4) Addressing health issues  5) Analyzing | **Results:**  *Barriers:*  1) Inability to take care of infant as much as desired  2) Balance between time spent at NICU and outside routine  3) Grief, frustration, anger, etc. affecting occupational performance  *Supports:*  1) Provided with options by professionals  2) “The little things”: changing diapers, taking temperature, etc.  3) Recognitions of “firsts” by professionals  **Significance of Findings:**  Neonatal occupational therapists can strengthen caregiver well-being and competence through parent-identified, observable, and non-observable co-occupations. |

| **Author/Year** | **Study Design, and**  **Risk of Bias** | **Participants, Inclusion Criteria, & Study Setting** | **Intervention and Control Groups** | **Outcome Measures** | **Results (Including significance of findings)** |
| --- | --- | --- | --- | --- | --- |
| 5.  **Dong et al., 2022**  DOI: [10.1111/jocn.16405](https://doi.org/10.1111/jocn.16405) | **Study Design**  Qualitative, Descriptive Study: Semi-Structured Interviews  **Risk of Bias**  Low | **Participants:**  n= 10 father**s**  **Inclusion Criteria**   - Fathers over the age of 18 - Speak English - At least one experience with kangaroo care   **Study Setting:**  NICU of Women and Children’s Hospital in South Australia | Narrative; Semi-Structured Interviews | ***Emerging Themes:***  **Positive Psychological Connection**  1) Anxiety  2) Calming  3) Infant-Parent Connection  4) Parenting Confidence  **Embracing Kangaroo Care**  1) Experiential learning  2) Restructured paternal caregiving role during NICU stay  **Challenges of Father-Infant Kangaroo Care**  1) Role Conflict  **Physical Discomfort**  1) Medical equipment and masculinity | **Results**  *Supports:*  1) Kangaroo care offers parents an alternative caretaking role in their baby’s life throughout the NICU stay  2) Kangaroo care is a supplemental parental occupation while the baby is in the NICU  **Significance of Findings:**  Kangaroo care has a positive impact in supporting parent-child co-occupation through fostering new parental roles and increasing confidence in parents. |

| **Author/Year** | **Study Design, and**  **Risk of Bias** | **Participants, Inclusion Criteria, & Study Setting** | **Intervention and Control Groups** | **Outcome Measures** | **Results (Including significance of findings)** |
| --- | --- | --- | --- | --- | --- |
| 6.  **Fraga et al., 2019**  DOI: [10.4322/2526-8910.ctoAO1125](https://doi.org/10.4322/2526-8910.ctoAO1125) | **Study Design:**  Qualitative, multiple case-study with Semi-Structured interviews and participant diaries  **Risk of Bias:**  Low | **Participants:**  n = 6 mothers  **Inclusion Criteria:**   - Infants born at 28 weeks or more - 4+ days stable in NICU - Literate - Primiparous - Mother staying in hospital with infant   **Study Setting:**  Philanthropic Institution in Belo Horizonte, Minas Gerias assisting patients of Unified Health System | Narrative; Semi-Structured Interviews | ***Emerging Themes:***  **Defining Mother’s Understanding of Motherhood:**  1) Caring for child  2) Establishing a relationship  **Motherhood in NICU Context**:  1) Negative Emotion  2) Barriers  3) Supportive Factors  **Development of Co-Occupations**:  1) New occupational roles as NICU parent | **Results:**  *Barriers:*  1) Medical technology  2) Lack of closeness  3) Sharing caretaking  4) Split between home and NICU  5) Medical restrictions  *Supports:*  1) Companionship/Support Groups  2) Spirituality  3) Personal Motivation  4) Establishing specific roles as caretaker (hand hygiene to protect baby)  5) Providing breast milk  6) Involvement in care  7) Kangaroo care  8) Establishing comfort occupations through voice/song  **Significance of Findings:**  Co-occupations can be created within the context of the NICU by balancing the limitations with supportive roles. |

| **Author/Year** | **Study Design, and**  **Risk of Bias** | **Participants, Inclusion Criteria, & Study Setting** | **Intervention and Control Groups** | **Outcome Measures** | **Results (Including significance of findings)** |
| --- | --- | --- | --- | --- | --- |
| 7.  **Gibbs et al., 2016**  DOI:  [10.1177/000841741562542](https://doi.org/10.1177/0008417415625421)1 | **Study Design:**  Qualitative, Descriptive Study with paradigmatic analysis of narratives; Semi-Structured Interviews  **Risk of Bias:**  Low | **Participants:**  n = 6 parents (3 couples)  **Inclusion criteria:**   - Being a parent of an infant born prematurely - Infant born less than 32 weeks’ gestation - Infant weighing less than 1,500 g birth weight - Infant requires invasive and noninvasive ventilation for a minimum of 7 days while in the NICU - Infant length of stay greater than 4 weeks - Having been discharged from the NICU 3 to 6 months prior, and - English speaking   **Study Setting:**  Level 3 NICU in Canada | Narrative; Semi-Structured Interview | ***Emerging Themes:***  1) Anticipating occupations versus reality  2) Needing emotional resilience  3) Working to reclaim the parental role  4) Navigating the NICU occupation–environment transactions  5) Building and maintaining relationships with staff  6) Revisioning the future | **Results:**  *Barriers:*  1) Role expectations not met  2) Emotional Demands/Stress  3) Occupational disruption  4) Medical technology/equipment (physical barrier)  5) NICU policies  6) Exclusion from healthcare rounds  7) Inconsistent advice  *Supports:*  1) Seeking alternative roles  2) Engagement in tube feeds  3) Modification of physical contact with infant  4) Breastfeeding  5) Peer supports  6) Positive communication with healthcare team  7) Encouragement from healthcare team  **Significance of Findings:**  An occupation-based practice approach is beneficial for parents of preterm infants in the NICU to find their parental role identity |

| **Author/Year** | **Study Design, and**  **Risk of Bias** | **Participants, Inclusion Criteria, & Study Setting** | **Intervention and Control Groups** | **Outcome Measures** | **Results (Including significance of findings)** |
| --- | --- | --- | --- | --- | --- |
| 8.  **Günay et al., 2021**  DOI: [10.1177/1054773820937479](https://doi.org/10.1177/1054773820937479) | **Study Design:**  Qualitative, Descriptive Study; Semi-Structured Interview  **Risk of Bias:**  Low | **Participants:**  n=12 fathers  **Inclusion Criteria:**  Father of infant that was:   - Birth weight > 1000g - Gestational age 27-36 weeks and postnatal age <28 days - Able to visit infant regularly - No serious health issues   **Study Setting:**  Level 3 NICU in Eastern Turkey | **Kangaroo Care:**   - Training on the benefits and application of kangaroo care was provided to fathers - Baby placed on fathers epigastric region 15-30 mins 2x day for 15 days   All participants participated in the intervention  Narrative: Semi-Structured Interview | ***Emerging Themes:***  (1) Emotions of being a father  (2) Confidence in fathering roles  (3) Happiness in the new father role | **Results:**  *Barriers:*  1) Fear of touching baby (before KC)  2) Fear of losing baby (before/ after KC)  3) Feelings of estrangement (before KC)  *Supports:*  1) Feeling warmth and scent  2) Feeling that baby belongs to them  3) Increased self-confidence  4) Felt responsibility  5) Increased happiness  6) Increased reciprocal calmness of infant and parent  **Significance of Findings:**  Healthcare professionals have the opportunity to strengthen parent-infant bonding and improve caregiver’s self-efficacy in parental roles through the intervention of Kangaroo Care. |

| **Author/Year** | **Study Design, and**  **Risk of Bias** | **Participants, Inclusion Criteria, & Study Setting** | **Intervention and Control Groups** | **Outcome Measures** | **Results (Including significance of findings)** |
| --- | --- | --- | --- | --- | --- |
| 9.  **Klawetter et al., 2019**  DOI: [10.1080/00981389.2019.1629152](https://dx.doi.org/10.1080/00981389.2019.1629152) | **Study Design:**  Qualitative, Descriptive Study; Semi-Structured Interviews  **Risk of Bias:**  Low | **Participants:**  n= 14 participants  **Inclusion Criteria:**   - English speaking mothers - Infants born less than 32 weeks and admitted to NICU at least 2 weeks - Infants aged 33-34 gestational age at start of study   **Study Setting:**  2 NICUs in US | Narrative; Semi-Structured Interviews | ***Emerging Themes:***  **Mother’s experience of engagement categorized as:**  1) Stress and trauma of NICU (medical, psychological, physical)  2) Barriers of engagement  3) Supports of engagement | **Results:**  *Barriers:*  1) Occupational disruption  2) Lack of medical knowledge for infant  3) Role conflicts (home and NICU)  4) Negative emotions  5) NICU Environment/Ergonomics  *Supports:*  1) Inclusion with healthcare team  2) Prior parenting experience  3) Companionship  4) Privacy in NICU  5) Well-designed NICU  6) Good healthcare team communication  7) Nurses advocating for parents  **Significance of Findings:**  The mother’s perspective provides an important understanding of how mothers can engage better in NICU co-occupations by having needs met by design and interaction in NICU. |

| **Author/Year** | **Study Design, and**  **Risk of Bias** | **Participants, Inclusion Criteria, & Study Setting** | **Intervention and Control Groups** | **Outcome Measures** | **Results (Including significance of findings)** |
| --- | --- | --- | --- | --- | --- |
| 10.  **Lilliesköld et al., 2022**  DOI:  [10.1016/j.jogn.2021.10.002](https://doi.org/10.1016/j.jogn.2021.10.002) | **Study Design:**  Qualitative, Descriptive Study; Semi-Structured Interview  **Risk of Bias:**  Low | **Participants:**  n= 6 parent couples  **Inclusion Criteria:**   - Parents participated in RCT that evaluated Immediate Parent-Infant Skin-to-skin contact (IPISTOSS) of preterm infant - Ability to speak English or Swedish   **Study Setting:**  Birth and Neonatal units in University hospital in Sweden | **IPISTOSS:**  Either parent participated in SSC the first 6 hours after birth   - All parents participated in intervention   Narrative; Semi-Structured Interview | ***Emerging Themes:***   1. Pathway to Connectedness 2. Just Being in a Vulnerable State 3. Creating a Safe Haven in an Unknown Terrain | **Results:**  *Barriers:*  1) Feelings of being an observer in infant’s care  2) Frightened by small and fragile infant (before SSC)  3) Insecure and scared of doing something wrong (before SSC)  4) Impaired by physical condition of mother  5) Inability of parents to express needs in SSC  *Supports:*  1) SSC increased feelings of connectedness  2) SSC felt it gave parents a role  3) Reciprocal calmness between parent and infant  4) Increased confidence of self as parent  5) Calmness and affirmations as staff made parents feel more prepared  **Significance of Findings:**  Early SSC can increase bond and elicit positive emotions between the parents and infants. This experience can be enhanced by the staff being available and exhibiting positive behaviors. |

| **Author/Year** | **Study Design, and**  **Risk of Bias** | **Participants, Inclusion Criteria, & Study Setting** | **Intervention and Control Groups** | **Outcome Measures** | **Results (Including significance of findings)** |
| --- | --- | --- | --- | --- | --- |
| 11.  **Maastrup et al., 2018**  DOI: [10.1111/scs.12478](https://doi.org/10.1111/scs.12478) | **Study Design:**  Qualitative, Descriptive Study; Semi-Structured Interviews  **Risk of Bias:**  Low | **Participants:**  n= 16 parents (3 couples)  **Inclusion Criteria:**   - Parents of a preterm infant(s) <28 weeks PMA   **Study Setting:**  Level 3 NICU in a Danish university hospital | Narrative; Semi-Structured Interviews | ***Emerging Themes:***  **1) Skin-to-Skin contact bonding is beneficial regardless of survival**   - Overcoming ambivalence through professional support and personal experience - Proximity creating parental feelings and an inner need to provide care - Feeling useful as a parent and realizing the importance of skin-to-skin contact | **Results:**  *Barriers:*  1) Ambivalence  2) Incubator is a barrier to feeling close to their infant  3) Conflicting thoughts and feelings about skin to skin from parents before being convinced it was safe  *Supports:*  1) Parents did not return to ambivalence once they had overcome it  2) Having real relationship when the infant was skin-to-skin, rather than having contact ‘through plexiglass’  **Significance of Findings:**  Skin to skin helps parents immediately move from feeling ambivalent to having a fundamental mutual need for skin to skin |

| **Author/Year** | **Study Design, and**  **Risk of Bias** | **Participants, Inclusion Criteria, & Study Setting** | **Intervention and Control Groups** | **Outcome Measures** | **Results (Including significance of findings)** |
| --- | --- | --- | --- | --- | --- |
| 12.  **Mäkelä et al., 2018**  DOI: [10.1016/j.midw.2018.04.003](https://doi.org/10.1016/j.midw.2018.04.003) | **Study Design:**  Qualitative, Descriptive Study; Journaling  **Risk of Bias:**  Low | **Participants:**  n= 23 parents (19 infants)  **Inclusion Criteria:**   - Parents - Able to read Finnish - Provided informed consent   **Study Setting:**  Level 3 NICU in Finland | Parents recorded their stories and experiences using the HAPPY smartphone app  Narrative; Journaling | ***Emerging Themes:***  Encompassing theme was the parents’ desire to be close and create a bond with their infant  1) Rollercoaster of closeness and separation  2) Bonding moments  3) Disrupted dyadic relationship | **Results:**  *Barriers:*  1) Physical distance  2) Separation from the infant  *Supports:*  1) Holding  2) Skin to skin  3) Participation in infant care  4) Breastfeeding  5) Spending time in the NICU and watching their infant  6) Infant reactions to parent’s care  7) Moments alone with their babies; as a family  8) NICU staff  **Significance of Findings:**  In order for parents and infants to bond in the NICU, they need to be physically close and have alone time. A peaceful environment will also support the bonding process. |

| **Author/Year** | **Study Design, and**  **Risk of Bias** | **Participants, Inclusion Criteria, & Study Setting** | **Intervention and Control Groups** | **Outcome Measures** | **Results (Including significance of findings)** |
| --- | --- | --- | --- | --- | --- |
| 13.  **Nelson & Bedford, 2016**  DOI:  [10.1016/j.pedn.2016.01.001](https://doi-org.libux.utmb.edu/10.1016/j.pedn.2016.01.001) | **Study Design**  Phenomenological Qualitative; Semi-Structured Interviews  **Risk of Bias:**  Low | **Participants:**  n= 7 Mothers  **Inclusion Criteria:**   - Infants were part of NIDCAP during NICU stay - Infants were born at 30 weeks or less.   **Study Setting:**  Level III NICU in Northeastern United States implementing the NIDCAP (Newborn Individualized Developmental Care and Assessment Program). | Narrative; Semi-Structured Interview | ***Emerging Themes:***  **Overarching Theme: Parenting with Permission**  1) Choosing to Participate  -Managing  -Settling  -Making Friends  2) Dealing with People  -Meeting needs  -Facing judgment  -Recognizing not everyone is on “board”  3) Coming to feel like a Mother  -Overcoming fear  -Gaining understanding  -Feeling empowered | **Results:**  *Barriers:*  1) Role Conflicts  2) NICU set-up  3) Poor Nurse-Parent Relationships  4) Restrictions of physical touch with infant  5) Fear of injuring or harming infant during caretaking activities  *Supports:*  1) Invitation to participate in infant caretaking (by nurse)  2) NIDCAP to empower and educate parents in role of caring for infant at different developmental stages  3) Single Infant Rooms (Family Privacy)  4) Positive Nurse-Parent Relationship  **Significance of Findings:** NIDCAP provides parents with “permission to parents” which empowers and allows them to be more involved in co-occupations of care. |

| **Author/Year** | **Study Design, and**  **Risk of Bias** | **Participants, Inclusion Criteria, & Study Setting** | **Intervention and Control Groups** | **Outcome Measures** | **Results (Including significance of findings)** |
| --- | --- | --- | --- | --- | --- |
| 14.  **Olsson et al., 2017**  DOI:  [10.1016/j.pedn.2017.03.004](https://doi.org/10.1016/j.pedn.2017.03.004) | **Study Design**  Descriptive Qualitative; Semi-Structured Interviews  **Risk of Bias:**  Low | **Participants:**  n= 20 Fathers  **Inclusion Criteria:**   - Variety of Fathers - Must have provided skin-to-skin at least once with their NICU infant   **Study Setting:**  1 County NICU and 1 University NICU in Sweden | Narrative; Semi-Structured Interview | ***Emerging Themes:***  1) Heart-Warming Experience: Closeness with Infant  2) Relieving Emotional Suffering:  Disconnect from NICU Stress  3) Rewarding Experiencing  4) Natural Instinct  5) A Learning Experience  6) Finding A Role  7) Improved Self Esteem  8) Feeling of Control  9) Supportive Environment  10) A Way of Knowing and Understanding  11) Important for Infant  12) Bonding Experience  13) Intimate Togetherness  14) Environment as an obstacle  15) Physical/Emotional Burden  16) Incongruence of Wishes and Demands  17) Uncertainty of Purpose and Skill in SSC  18) Fear of Hurting Infants  19) Feeling Insufficient for Family | **Results:**  *Barriers:*  1) Medical technology  2) Role conflict/strain  3) Fear of harming infant  *Supports:*  1) Skin to Skin Care allows Fathers to feel a responsibility in caring for the infant  2) Encouragement for participation by nursing staff  **Significance of Findings:**  Skin to skin contact provides a new opportunity for parents to create a role and co-occupation in comfort and caretaking of infant. SSC also decreases negative emotions and increases confidence in parenting occupations. |

| **Author/Year** | **Study Design, and**  **Risk of Bias** | **Participants, Inclusion Criteria, & Study Setting** | **Intervention and Control Groups** | **Outcome Measures** | **Results (Including significance of findings)** |
| --- | --- | --- | --- | --- | --- |
| 15.  **Ringham et al., 2022**  DOI: [10.1097/ANC.0000000000000984](https://dx.doi.org/10.1097/ANC.0000000000000984) | **Study Design**  Qualitative; Ethnography  **Risk of Bias:**  Low | **Participants:**  n= 614 parents  **Inclusion Criteria:**   - Mothers in Level II NICUs either receiving standard care or FICare - Mothers who wrote substantial reflections in journal (101 were included in analysis)   **Study Setting:**  10 Level II NICUs in Alberta, Canada | 308 participants had infants receiving FICare (Family-Integrated Care)  306 participants had infants receiving standard care in the NICU  101 journals were included in study | ***Emerging Themes:***  1) Feeding policies and practice  2) Rollercoaster of emotions  3) The work of mothering in the NICU | **Results:**  *Barriers:*  1) NICU Feeding Guidelines  2) Lack of opinion allowed in infant feeding schedule  3) Difficulties with breastfeeding  4) Role conflict and strain  5) Emotional intensity  6) NICU rules  *Supports:*  1) Focus on feeding participation and breast milk expression  **Significance of Findings:**  Family-Integrated Care encourages increased participation by parents in feeding and other caretaking roles which provide modified occupations for Mothers’ to fulfill co-occupations to the best ability. Standard NICU care creates a loss of control in parents between guidelines and rules. |

| **Author/Year** | **Study Design, and**  **Risk of Bias** | **Participants, Inclusion Criteria, & Study Setting** | **Intervention and Control Groups** | **Outcome Measures** | **Results (Including significance of findings)** |
| --- | --- | --- | --- | --- | --- |
| 16.  **Santos et al., 2019**  DOI:  [10.1590/1980-265X-TCE-2018-0394](https://doi.org/10.1590/1980-265X-TCE-2018-0394) | **Study Design**  Descriptive Qualitative Study; Semi-Structured Interview  **Risk of Bias:**  Moderate | **Participants:**  n= 23 mothers  **Inclusion Criteria:**  Mothers present at NICU on day of interview  **Study Setting:**  Public NICU in Brazil | Narrative; Semi-Structured Interviews | **Emerging Themes:**  1) Learning Needs   - Inability to perform mothering role - Maternal Insecurity   2) Barriers to Bonding  3) Tension in Mother Role  4) Maternal Feelings  5) Maternal Stress | **Results:**  *Barriers:*  1) Medical technology  2) Limited role in caretaking responsibility  3) Physical separation from infant and limited touch  4) Lack of knowledge for fragile infant caregiving  5) Lack of parental permission  6) Fear of hurting infant  **Significance of Findings:**  Barriers to infant-mother co-occupations include the abundance of medical equipment, separation and lack of physical touch with infants, and fear of interacting with infants due to limited knowledge on fragile infant caretaking. |

| **Author/Year** | **Study Design, and**  **Risk of Bias** | **Participants, Inclusion Criteria, & Study Setting** | **Intervention and Control Groups** | **Outcome Measures** | **Results (Including significance of findings)** |
| --- | --- | --- | --- | --- | --- |
| 17.  **Spence et al., 2023**  DOI: [10.3390/ijerph20116050](https://doi.org/10.3390/ijerph20116050) | **Study Design:**  Qualitative, Phenomenological Study; Semi-Structured Interview  **Risk of Bias:**  Low | **Participants:**  n= 12 parents of very preterm infants  **Inclusion Criteria:**   - Infant born prior to 29 weeks’ gestation - In SPEEDI-2 study - Level 3 or 4 NICU at one of three hospitals in Virginia - English speaking - Lived in 60 mile radius of hospital     **Study Setting:**  Level 3 or 4 NICU in Virginia | Narrative: Semi-Structured Interviews | ***Areas of Analysis:***  1) Experiences while in NICU  2) Support Impacting Experience while in NICU  3) Impact of COVID-19 on Parent Experience while in NICU  4) Transition from NICU to Home  5) First Few Weeks at Home  6) Impact of COVID-19 on Transition | **Results:**  *Barriers*:  1) Could not touch infant for 3 weeks  2) Lack of bonding  3) Lack of knowledge of medical concerns  4) Felt NICU staff was parenting  5) Lack of ability to participate in care routines  6) Lack of parent-staff communication  7) Transportation, distance, family obligations  8) Lack of connection with other parents  9) Restricted # of visitors (COVID)  10) Anxiety about going home (logistics of feeding & positioning)  *Supports:*  1) Engaging NICU staff  2) Early support from a multidisciplinary team (counselor, social work, etc.)  **Significance of Findings:**  Involving parents in care routines, frequent communication, education, and psychosocial support can increase NICU parents' self-efficacy and reduce stress. |

| **Author/Year** | **Study Design, and**  **Risk of Bias** | **Participants, Inclusion Criteria, & Study Setting** | **Intervention and Control Groups** | **Outcome Measures** | **Results (Including significance of findings)** |
| --- | --- | --- | --- | --- | --- |
| 18.  **Spinelli et al., 2016**  DOI:  [10.1080/08870446.2015.1088015](https://doi.org/10.1080/08870446.2015.1088015) | **Study Design**  Descriptive Qualitative Study; Semi-Structured Interview  **Risk of Bias:**  Low | **Participants:**  n= 30 mothers  **Inclusion Criteria:**   - Infants born between 24- and 34-weeks’ gestation - Infant in stable condition - Consent from both parents - Infant has no known congenital or neurological impairments - Mothers at least 18 years old and speak Italian   **Study Setting:**  NICU in Northern Italy | Narrative; Semi-Structured Interviews | ***Emerging Themes:***  1) Disconnection from Child  2) Perception of Maternal Inadequacy  3) Loss of Parental Role  4) Temporal Suspension | **Results:**  *Barriers:*  1) Physical separation from child  2) Supervision of medical staff in interactions with child  3) NICU Environment  4) Medical technology  5) Lack of parental permission  6) Lack of physical contact with infant  7) Lack of privacy  8) Poor relationships with nursing and providers  **Significance of Findings:**  Parents to infants being treated in the NICU are deprived of a smooth transition to motherhood due to physical separation, lack of control, environmental barriers in the NICU, and poor nursing-parent interactions. |

| **Author/Year** | **Study Design, and**  **Risk of Bias** | **Participants, Inclusion Criteria, & Study Setting** | **Intervention and Control Groups** | **Outcome Measures** | **Results (Including significance of findings)** |
| --- | --- | --- | --- | --- | --- |
| 19.  **Treherne et al., 2017**  DOI: [10.1016/j.jogn.2017.07.005](https://doi.org/10.1016/j.jogn.2017.07.005) | **Study Design:**  Qualitative, Descriptive: Recorded Verbal Journal  **Risk of Bias:**  Low | **Participants:**  n= 22 parents  **Inclusion Criteria:**  Parent of infant who was:   - Preterm infant (<37 weeks gestational age) - Stable medical condition of infant - Able to read English or French - Able to provide informed consent   **Study Setting:**  Urban Level 3 NICU in Canada | Narrative; Recorded Verbal Journal | Data verbally collected on smart phone application Handy Application to Promote Preterm infant happY-life (HAPPY) when parents felt close to or separate from their infant.  Verbal description of:   - How they were feeling - What was happening   Who was involved | **Results:**  *Barriers:*   - Restriction of autonomy due to schedule - Separate when pumping without infant - Inadequate when nurse stepped in during alone time - Guilt, anxiety, and sadness when leaving hospital to take care of self or other responsibilities - Feelings of separateness in open environment due to lack of privacy and artificiality - Wires interfered with bath time   *Supports:*   - Empowerment when directly involved in care and acted independently (temperature, diaper, feeding) - Closeness when feeding, holding, interacting - Closeness when staff made parent feel like a part of the team (transparency, interdisciplinary rounds, charts) - Spending time alone without nurse - Closeness when NICU was quiet   **Significance of Findings:**  Parents felt closeness when they were given opportunities to make decisions and autonomy in the activities they participated in with their infants. |

| **Author/Year** | **Study Design, and**  **Risk of Bias** | **Participants, Inclusion Criteria, & Study Setting** | **Intervention and Control Groups** | **Outcome Measures** | **Results (Including significance of findings)** |
| --- | --- | --- | --- | --- | --- |
| 20.  **Yu et al., 2020**  DOI:  [10.1016/j.pedn.2019.11.002](https://doi.org/10.1016/j.pedn.2019.11.002) | **Study Design**  Descriptive Qualitative Study; Semi-Structured Interview  **Risk of Bias:**  Low | **Participants:**  n= 15 parents  **Inclusion Criteria:**   - 18 or older - Parent to a preterm infant - Preterm infant admitted to NICU for at least 7 days - Infant condition was stable - Participant would be primary caregiver after NICU discharge   **Study Setting:**  Specialized NICU in Central China | Narrative; Semi-Structured Interview | ***Emerging Themes:***  1) Mixed Emotional Experiences  2) Separation from Infants  3) Perceived Incompetence in taking care of Preterm Infant  4) Obtained support through various sources  5) Desired more from healthcare professionals | **Results:**  *Barriers:*  1) Emotional Experience (fear, anxiety, worry, guilt) from separation and feelings of incompetence  2) Lack of experience with infant (low confidence)  3) Physical separation from infant and NICU policies  4) Poor communication with health care team  *Supports:*  1) Family education classes for infant caretaking  2) Virtual connection with infant  **Significance of Findings:**  The experience of parenting an infant in the NICU is incredibly complex for new parents. Many experiences a range of emotions due to lack of involvement in co-occupations often by lack of caregiving opportunity, physical separation, and poor communication. However, many of these same concerns were alleviated by supporting connection with technology or educating families. |
