# Supplementary material for: Parent Perspectives of Co-Occupations in Neonatal Intensive Care: A Thematic Review of Barriers and Supports
Source: OTJR (Thorofare N J). 2024 Aug 19;45(3):378–87. doi: 10.1177/15394492241271220 (PMC12130602; doi:10.1177/15394492241271220)
Supplement: sj-docx-2-otj-10.1177_15394492241271220 – Supplemental material for Parent Perspectives of Co-Occupations in Neonatal Intensive Care: A Thematic Review of Barriers and Supports [file sj-docx-2-otj-10.1177_15394492241271220.docx]

***Appendix B***

CASP Qualitative Checklist: Critical Appraisal Skills Programme

*Critical Appraisal Skills Programme (2022). CASP Qualitative Checklist. Available at:*<https://casp-uk.net/images/checklist/documents/CASP-Qualitative-Studies-Checklist/CASP-Qualitative-Checklist-2018_fillable_form.pdf> *Accessed: 07/14/2023.*

|  | **Was there a clear statement of the aims of the research?** | **Is a qualitative methodology appropriate?** | **Was the research design appropriate to address the aims for the research** | **Was the recruitment strategy appropriate?** | **Was the data collected in a way that addressed the research issue?** | **Has the relationship between research and participants been adequately considered?** | **Have ethical issues been taken into consideration?** | **Was the data analysis sufficiently rigorous?** | **Is there a clear statement of findings?** | **How valuable is this research?** |
| --- | --- | --- | --- | --- | --- | --- | --- | --- | --- | --- |
| Antinora et al., 2023 | Yes | Yes | Yes | Yes | Yes | Not Clear | Not Clear | Yes | Yes | Shows the need for role adaptations to provide parents the opportunity to engage in co-occupations, such as using technology to bridge distance between infants and parents. |
| Bonner et al., 2017 | Yes | Yes | Yes | Yes | Yes | Yes | Yes | Yes | Yes | Shows that wires impact the implementation of kangaroo care and thus have a negative affect on parent-infant bonding. Also shows that parents and nurses are positively receptive to a wireless monitoring system. |
| Campbell-Yeo et al., 2021 | Yes | Yes | Yes | Yes | Yes | Yes | Yes | Unclear | Yes | Shows increase in participation of Father’s who care for infants staying in single-family rooms. Additionally, single-family rooms were associated with more breast milk expression by mothers. |
| Cardin, 2020 | Yes | Yes | Yes | Yes | Yes | Yes | Yes | Yes | Yes | NICU OTs have the opportunity to increase confidence and bonding through parent-identified co-occupations, as well as serve as a liaison and advocate between the parent and professional team. |
| Dong et al., 2022 | Yes | Yes | Yes | Yes | Yes | Yes | Yes | Yes | Yes | NICU Fathers report an increase in parenting participation and confidence following engagement in Kangaroo Care. |
| Fraga et al., 2009 | Yes | Yes | Yes | Yes | Yes | Yes | Yes | Yes | Yes | Explains specific barriers and supports in narrative form.  Gives personal perspective to review. |
| Gibbs et al., 2016 | Yes | Yes | Yes | Yes | Yes | Yes | Yes | Yes | Yes | The alteration of role expectation is the largest barrier of NICU co-occupations but can be remedied with other parenting co-occupation roles. |
| Günay et al., 2021 | Yes | Yes | Yes | Yes | Yes | Yes | Yes | Yes | Yes | Demonstrates the significance of implementation of kangaroo care for fathers for eliciting positive emotions and strengthening parent-infant bonds. |
| Klawetter et al., 2019 | Yes | Yes | Yes | Yes | Yes | Yes | Yes | Yes | Yes | Provides insight on a mother’s perspective in their ability to engage in parenting occupations while in NICU. |
| Lilliesköld et al., 2022 | Yes | Yes | Yes | Yes | Yes | Yes | Yes | Yes | Yes | Immediate SSC elicits positive emotions and strengthens bonding between parent and infants. Neonatal staff can enhance experience by addressing parent needs and being available. |
| Maastrup et al., 2018 | Yes | Yes | Yes | Yes | Yes | Yes | Yes | Yes | Yes | Provides insight on how skin to skin helps parents immediately move from feeling ambivalent to having a fundamental mutual need for skin to skin. |
| Mäkelä et al., 2018 | Yes | Yes | Yes | Yes | Yes | Yes | Yes | Yes | Yes | Show how much of an impact physical distance can have on the relationship between a parent and infant. This article demonstrates how a stay in the NICU is not a linear process for the bond between parent and infant. |
| Nelson & Bedford, 2016 | Yes | Yes | Yes | Yes | Yes | Yes | Yes | Yes | Yes | This study explains that the NIDCAP program implementation provides parents with a “permission to parent” which is shown to increase engagement in infant-parent co-occupations in the NICU. |
| Olsson et al., 2017 | Yes | Yes | Yes | Yes | Yes | Yes | Yes | Yes | Yes | Skin to skin contact provides a unique co-occupations for parents to engage in with their infants as a method of comfort and caretaking. It provides some responsibility. |
| Ringham et al., 2022 | Yes | Yes | Yes | Yes | Yes | Yes | Yes | Yes | Yes | Mothers whose infants received FICare had increased participation and motivation to participate in activities such as feedings. While standard care Mothers identified barriers such as loss of control in NICU practices and rules with lack of participation. |
| Santos et al., 2019 | Yes | Yes | Yes | Not Clear | Yes | Not Clear | Not Clear | Yes | Yes | Several barriers can be identified by mothers of NICU infants in terms of bonding and motherhood role impairments limitations. |
| Spence et al., 2023 | Yes | Yes | Yes | Yes | Yes | Yes | Yes | Yes | Yes | Explains the deeper impact of physical separation with infants and lack of participation in caregiving as an immense barrier. In studying extremes during covid, further evidence of the role of physical and role separation in disturbing co-occupations. |
| Spinelli et al., 2016 | Yes | Yes | Yes | Yes | Yes | Yes | Yes | Yes | Yes | The transition to motherhood is disrupted when a newborn requires a NICU stay. This creates disconnection and feelings of powerlessness which deprives parents of co-occupations with their infant. |
| Treherne et al., 2017 | Yes | Yes | Yes | Yes | Yes | Yes | Yes | Yes | Yes | Significance of opportunities of autonomy and environmental factors that made parents feel close or separated from their infant. |
| Yu et al., 2020 | Yes | Yes | Yes | Yes | Yes | Yes | Yes | Yes | Yes | Shows importance of connection and education for new parents while showing how lack of engagement in co-occupations with infants can be distressing for new parents. |

References

Antinora, C., Taylor-Ducharme, S., Asselin, S., Jacquet, C., Ducharme-Roy, D., Wazneh, L., Morrison, S., Sinclair, K., Duby, J. (2023). Neoconnect: the design, implementation, and impact of a virtual family-centered NICU program.. *Journal of Perinatal & Neonatal Nursing*, *37*(1), 61-67. <https://dx.doi.org/10.1097/JPN.0000000000000698>

Bonner, O., Beardsall, K., Crilly, N., & Lasenby, J. (2017). 'There were more wires than him': the potential for wireless patient monitoring in neonatal intensive care. *BMJ Innovations*, *3*(1), 12–18. <https://doi.org/10.1136/bmjinnov-2016-000145>

Campbell-Yeo, M., Kim, T., Disher, T., Richardson, B., Dol, J., Bishop, T., Delahunty-Pike, A., Dorling, J., Glover, M., Inglis, D., Johnson, T., Macmillan, D., Mcgrath, P., Monaghan, J., Orovec, A., Simpson, D., Skinner, N., Whitehead, L., & Wozney, L.(2021). Do single-family rooms increase parental presence, involvement, and maternal well-being in neonatal intensive care?. *The Journal of Perinatal & Neonatal Nursing*, *35*(4), 350–361. <https://doi.org/10.1097/JPN.0000000000000600>

Cardin, A. (2020). Parents' perspectives: An expanded view of occupational and co-occupational performance in the neonatal intensive care unit. *The American Journal of Occupational Therapy*, *74*(2), 1-12. <https://doi.org/10.5014/ajot.2020.034827>

Craig, J., Carroll, S., Ludwig, S., & Sturdivant, C. (2018). Occupational therapy's role in the neonatal intensive care unit. *The American Journal of Occupational Therapy, 72*, 1-9. <https://doi.org/10.5014/ajot.2018.72S204>

Critical Appraisal Skills Programme (2018). CASP qualitative studies checklist. *CASP Checklists.* <https://casp-uk.net/images/checklist/documents/CASP-Qualitative-Studies-Checklist/CASP-Qualitative-Checklist-2018_fillable_form.pdf>

Dong, Q., Steen, M., Wepa, D., & Eden, A. (2022). Exploratory study of fathers providing kangaroo care in a neonatal intensive care unit. *Journal of Clinical Nursing*. <https://doi.org/10.1111/jocn.16405>

Fraga, E., Dittz. E., & Machado, L. (2019). The construction of maternal co-occupation in the neonatal intensive care unit. *Brazilian Journal of Occupational Therapy*, *27*(1): 92-104. doi:10.4322/2526-8910.ctoAO1125

Gibbs, D., Boshoff, K., & Stanley, M. (2016). The acquisition of parenting occupations in neonatal intensive care: A preliminary perspective. *Canadian Journal of Occupational Therapy*, 83(2), 91–102. <https://doi.org/10.1177/0008417415625421>

Günay, U., & Coşkun Şimşek, D. (2021). Emotions and experience of fathers applying kangaroo care in the eastern anatolia region of turkey: A qualitative study. *Clinical Nursing Research*, *30*(6), 840–846. <https://doi.org/10.1177/1054773820937479>

Harer M. & Moreno M. (2019). What parents need to know about the neonatal intensive care unit. *The Journal of the American Medical Association Pediatrics, 173*(5), 508. doi:10.1001/jamapediatrics.2019.0256

Klawetter, S., Neu, M., Roybal, K., Greenfield, J., Scott, J., Hwang, S. (2019). Mothering in the nicu: a qualitative exploration of maternal engagement. *Social Work in Health Care*, *58*(8), 746-763. <https://dx.doi.org/10.1080/00981389.2019.1629152>

Lilliesköld, S., Zwedberg, S., Linnér, A., & Jonas, W. (2022). Parents' experiences of immediate skin-to-skin contact after the birth of their very preterm neonates. *Journal of Obstetric, Gynecologic, and Neonatal Nursing*, *51*(1), 53–64. <https://doi.org/10.1016/j.jogn.2021.10.002>

Maastrup, R., Weis, J., Engsig, A., Johannsen, K., & Zoffmann, V. (2018). 'Now she has become my daughter': Parents' early experiences of skin-to-skin contact with extremely preterm infants. *Scandinavian Journal of Caring Sciences*, *32*(2), 545–553. <https://doi.org/10.1111/scs.12478>

Mäkelä, H., Axelin, A., Feeley, N., & Niela-Vilén, H. (2018). Clinging to closeness: The parental view on developing a close bond with their infants in a NICU. *Midwifery*, *62*, 183–188. <https://doi.org/10.1016/j.midw.2018.04.003>

Nelson, A., & Bedford, P. (2016). Mothering a preterm infant receiving NIDCAP care in a level III newborn intensive care unit. *Journal of Pediatric Nursing*, *31*(4), 271–282. [https://doi.org/10.1016/j.pedn.2016.01.001](https://doi-org.libux.utmb.edu/10.1016/j.pedn.2016.01.001)

Olsson, E., Eriksson, M., & Anderzén-Carlsson, A. (2017). Skin-to-skin contact facilitates more equal parenthood - A qualitative study from fathers' perspective. *Journal of Pediatric Nursing*, *34*, 2–9. <https://doi.org/10.1016/j.pedn.2017.03.004>

Page M., McKenzie J., Bossuyt P., Boutron I., Hoffmann T., Mulrow C., et al. (2021). The PRISMA 2020 statement: An updated guideline for reporting systematic reviews. *British Medical Journal, 372*(71). doi: 10.1136/bmj.n71

Pierce, D. (2009). Co‐occupation: The challenges of defining concepts original to occupational science. *Journal of Occupational Science*, *16*(3), 203–207. https://doi.org/10.1080/14427591.2009.9686663

Price, P., & Miner, S. (2009). Extraordinarily ordinary moments of co-occupation in a neonatal intensive care unit. *Occupational Therapy Journal of Research, 29*, 72–78. https://doi.org/10.3928/15394492-20090301-04

Ringham, C., McNeil, D., Benzies, K. M. (2022). The work of mothering in the NICU: A critical analysis of alberta family integrated care parent journals. *Advances in Neonatal Care*, *22*(4), E112-E119. <https://dx.doi.org/10.1097/ANC.0000000000000984>

Santos, A., Rodrigues, L., Santos, M., Sousa, G., Viana, M., & Chaves, E. (2019). Maternal role during child’s hospitalization in the neonatal intensive therapy unit. *Texto & Contexto - Enfermagem*, *28*. <https://doi.org/10.1590/1980-265X-TCE-2018-0394>

Spence, C., Stuyvenberg, C., Kane, A., Burnsed, J., Dusing, S. (2023). Parent experiences in the NICU and transition to home. *International Journal of Environmental Research & Public Health*, *20*(11). <https://dx.doi.org/10.3390/ijerph20116050>

Spinelli, M., Frigerio, A., Montali, L., Fasolo, M., Spada, M., & Mangili, G. (2016). 'I still have difficulties feeling like a mother': The transition to motherhood of preterm infants' mothers. *Psychology & Health*, *31*(2), 184–204. <https://doi.org/10.1080/08870446.2015.1088015>

Treherne, S., Feeley, N., Charbonneau, L., & Axelin, A. (2017). Parents' perspectives of closeness and separation with their preterm infants in the NICU. *Journal of Obstetric, Gynecologic, and Neonatal Nursing*, *46*(5), 737–747. <https://doi.org/10.1016/j.jogn.2017.07.005>

Yu, X., Zhang, J., & Yuan, L. (2020). Chinese parents' lived experiences of having preterm infants in NICU: A qualitative study. *Journal of Pediatric Nursing*, *50*, 48–54. <https://doi.org/10.1016/j.pedn.2019.11.002>
